# Reducing the burden of surgical site infections in low and middle-income countries: challenges and recommendations

**DOI:** 10.1097/MS9.0000000000003473

**Published:** 2025-06-13

**Authors:** Muhammad Fawad Tahir, Sanila Mughal, Amna Nadeem, Maimoona Khan, Ramish Hannat, Mohammed Hammad Jaber Amin

**Affiliations:** a HBS Medical and Dental College, Islamabad, Pakistan; b Dow University of Health Sciences, Karachi, Pakistan; c The University of Faisalabad, Faisalabad, Pakistan; d Punjab Medical College, Faisalabad, Pakistan; e Services Institute of Medical Sciences, Lahore, Pakistan; f Alzaiem Alazhari University Faculty of Medicine, Khartoum, Sudan

## Abstract

Surgical site infections (SSIs) are a significant and growing challenge in surgical care, particularly in low- and middle-income countries (LMICs), where limited resources and inadequate infection control amplify their impact. Affecting up to 25% of surgical patients in some regions, SSIs lead to prolonged hospital stays, increased healthcare costs, and substantial patient suffering. Multidrug-resistant pathogens and inadequate adherence to global guidelines exacerbate the issue, highlighting the need for tailored interventions. Innovative approaches, such as active surveillance programs, routine changes in surgical practices, and enhanced patient education, have shown promise in reducing SSI rates. By prioritizing infection prevention strategies, resource optimization, and collaborative efforts between healthcare providers and policymakers, LMICs can significantly improve surgical outcomes and reduce the burden of SSIs.

Surgical site infections (SSIs) are a serious and common complication of surgical procedures leading to pain, disability, poor wound healing with risk of wound breakdown and hernia, prolonged recovery times, psychological challenges, and high resource utilization in the affected patients. The SSIs have been increasing over time, with the highest incidence during the COVID-19 pandemic. According to the Lancet Commission on Global Surgery, 313 million surgical procedures are performed worldwide annually^[^1^]^. SSIs are a serious and common complication of surgical procedures leading to pain, disability, poor wound healing with risk of wound breakdown and hernia, prolonged recovery times, psychological challenges, and high resource utilization in the affected patients. The SSIs have been increasing over time, with the highest incidence during the COVID-19 pandemic. These infections vary according to the type of procedure, but the highest risk is observed for orthopedic procedures followed by cardiac and intra-abdominal surgeries. The most common pathogens to cause SSIs are *Staphylococcus aureus* and *Escherichia coli*. The emergence of multidrug-resistant pathogens is one of the reasons for the increasing incidence of SSIs.

The global pooled incidence of SSIs is 2.5%. However, the incidence varies by region and country and is higher in developing countries than in the developed countries^[^2^]^.The incidence of SSIs in the United States is 2.6%, while in the African region, it is 7.2%, Sub-Saharan Africa 11-18%, and Ethiopia 12.5-25.22%, which are significantly higher^[^3^]^.

The 30-day all-cause mortality rate after surgery, often used as an indicator of the effectiveness of surgical care systems, accounts for 4.2 million deaths annually. Half of these fatalities occur in low- and middle-income countries (LMIC), making this the third leading cause of death worldwide, following ischemic heart disease and stroke^[^1^]^.

Since SSIs are a significant concern for patients undergoing surgical procedures in Pakistan, their impact on patient outcomes and healthcare costs cannot be ignored. A study reports that the incidence of SSI remains higher in low-Human development index (HDI) countries than in middle-HDI or high-HDI countries, with nearly 40% of patients undergoing the word dirty surgery in low-HDI settings developed SSI. Furthermore, it has been postulated that patients who develop SSI also tend to have more extended hospital stays, with their stay being three times longer than patients who do not develop SSI. Nevertheless, these findings suggest that efforts to reduce the incidence of SSI should focus on improving surgical safety and infection control measures, particularly in low-HDI countries where the problem is most severe. However, the lack of facilities and awareness in hospitals in Pakistan has hindered the implementation of these measures.

The rapidly increasing prevalence of SSI in LMICs requires novel approaches to overcome the disease burden caused by SSIs. To make the guidelines of Centres for Disease Control and Prevention (CDC) and World Health Organization (WHO) workable in low-income countries, the first step is the collaboration between healthcare professionals and patients. Educating patients about the seriousness of their situation and providing counseling can be very beneficial. It is important to advise patients to shower before surgery and to avoid using razor blades for hair removal at the surgical site. Health education plays a crucial role in this process. In areas where load shedding is a problem, alternative energy sources should always be available to maintain the operating room temperature between 21°C and 23°C. There is a dire need of better environmental hygiene and efficient infection control practices in public hospitals. Furthermore, it should be ensured that the attire of the surgeons and healthcare workers should be limited to the room. In teaching hospitals, a certain number of students should be allowed to observe in the operation theatre, and they must be provided with disposable surgical gowns, head and shoe covers to avoid contamination of the operation theatre.

In countries such as Pakistan and India, where resources are limited, the issue of SSIs can be addressed by conducting regular root cause analyses at the hospital level after each SSI case. Based on these analyses, quality improvement projects should be developed and implemented. Additionally, a cost analysis and an evaluation of the effectiveness of each intervention should be performed. This will help policymakers invest in the most effective and cost-efficient solutions. Apart from that, each case of SSI should be discussed at the morbidity and mortality (M&M) conferences in the hospitals to provide the clinicians with an opportunity to discuss errors and adverse events openly, review care standards, and make changes.

A study conducted in Ghana assessed the impact of a 30-day active surveillance program for SSIs. This program involved providing a wound card to post-operative patients and conducting periodic telephone follow-ups. This simple intervention required an additional investment of only $18.47 per patient but significantly reduced SSI morbidity and mortality by approximately one-third. As a result, patient costs decreased by nearly 12%, hospital costs dropped by about 25%, and the length of stay associated with SSIs was reduced by 33%. Implementing this strategy in other low- and middle-income countries (LMICs) could facilitate early detection and management of SSIs, shorten the length of hospital stays, and help address the issue of limited hospital bed capacity, creating opportunities to admit and treat more surgical patients effectively^[^4^]^.

Furthermore, in a trial conducted by The GlobalSurg Collaborative, it has been reported that organisms resistant to prophylactic antibiotics account for a significant percentage of SSIs, with LMICs bearing the heaviest load. About 35.9% of the patients in LMICS developed SSIs, compared to 16.6% in high-HDI and 19.8% in middle-HDI countries. This study also determined that antibiotic course in patients from low-income countries is longer than in high-income countries^[^5^]^. In this era of antibiotic resistance, rather than administering the previously universally accepted antibiotic prophylaxis, we should allocate funding to identify which antibiotics are currently effective in targeting nosocomial and SSIs in a particular population.

Various trials have been conducted to evaluate the recommendations of the Centres for Disease Control and Prevention (CDC) and the World Health Organization (WHO). One such trial is the FALCON trial, which was conducted in 54 hospitals across 7 low- and middle-income countries (Benin, Ghana, India, Mexico, Nigeria, Rwanda and South Africa. This trial demonstrated that neither 2% alcoholic chlorhexidine nor triclosan-coated sutures had a beneficial effect on reducing SSI at day 30, whether in clean-contaminated or contaminated and dirty surgical procedures in low- and middle-income countries (LMICs). Globally, we need more trials specifically focused on LMICs to determine whether the recommendations from the WHO and CDC should be reconsidered in these regions to conserve resources^[^6^]^. Whereas the ChEETAh trial found that routine change of gloves and instruments before abdominal wound closure resulted in a 13% reduction in surgical site infection (SSI) rate 30 days after surgery, compared to the control group. It is worth noting that this reduction in SSI was observed across a wide range of hospitals, including both large, tertiary-level hospitals with advanced perioperative services and small, rural hospitals with fewer resources^[^7^]^.

In conclusion, implementing infection control measures and proper sterilization techniques is essential in reducing the incidence of SSI in LMICs. The public healthcare authorities should prioritize the formation of sterilization committees and awareness programs to promote the safety and well-being of patients. We are hopeful that implementing the above suggestions in public and private sectors can help significantly decrease the prevalence of SSIs in LMICs, thereby paving the hope to reduce the burden of healthcare-associated costs and improve the postoperative care of the patients.

## Data Availability

No data was generated.
